# A Case Report of Refractory Graft-Versus-Host Disease Colitis Managed With Robotic Total Abdominal Colectomy

**DOI:** 10.7759/cureus.57829

**Published:** 2024-04-08

**Authors:** Olivia Ziegler, Neekita R Jikaria, Joseph Cioccio, Jeffery S Scow

**Affiliations:** 1 General Surgery, Penn State Health Milton S. Hershey Medical Center, Hershey, USA; 2 Hematology and Oncology, Penn State Cancer Institute, Hershey, USA; 3 Colorectal Surgery, Penn State Health Milton S. Hershey Medical Center, Hershey, USA

**Keywords:** acute graft-versus-host disease, gastrointestinal graft-versus-host disease, allogeneic stem cell transplant recipients, colon and rectal surgery, total abdominal colectomy

## Abstract

Graft-versus-host disease (GvHD) is a common complication following hematopoietic stem cell transplant (HSCT) and has protean manifestations. It results from the activation of transplanted T lymphocytes against the HLA antigens of recipient cells, resulting in tissue destruction. The most commonly involved sites of acute GvHD are the skin and gut, with high mortality reported in the latter.

Historically, surgery for gut GvHD has been reserved for those with frank perforations or uncontrolled hemorrhage. Here, we present a case of steroid and ruxolitinib refractory colonic GvHD in a 41-year-old female, which was ultimately managed with robotic-assisted total abdominal colectomy with resolution of enteric symptoms. This case highlights the role of surgical management in gut GvHD in patients who are refractory to the growing arsenal of immunomodulating agents. Given the rarity of surgical intervention in this population, more data are needed to minimize morbidity in this setting.

## Introduction

Gut graft-versus-host disease (GvHD) may involve the upper or lower GI tract, the liver, or combinations of these sites [1]. Its diagnosis and severity staging are based on clinical features, namely, watery diarrhea exceeding 1,500 mL per day signaling grade III gut GvHD and the development of ileus, abdominal pain, or bloody diarrhea signifying grade IV [2]. Diagnosis may be aided by endoscopy or histopathology to rule out related other entities, such as CMV colitis, given the non-specific nature of symptoms in an immunosuppressed patient [3,4].

The pathogenesis of gut GvHD is incompletely understood; immune dysregulation and damage from the conditioning regimen appear to disturb the intestinal epithelium [5], while aberrations in the gut microbiome may play a role in pathogenesis, and the degree of dysbiosis appears to correlate with long-term outcomes [6].

While skin GvHD is often sensitive to steroids and may even be treated topically, gut GvHD results in mortality in around half of cases [4,7] and presents unique treatment challenges [8]. IV and enteric-targeted corticosteroids represent the mainstay of initial treatment; however, almost 60% of patients are refractory to steroid therapy [9]. Higher rates of steroid refractory disease are observed in those with lower GI involvement, which, compared to cutaneous or other GI site involvement, confers a worse prognosis, as does steroid refractoriness alone [10]. In individuals with steroid refractory GvHD, the standard of care since 2019 has been to offer ruxolitinib (a JAK1/2 inhibitor), which leads to durable responses at eight weeks in 39% of patients [11]. Unfortunately, the overall survival in patients who are refractory to ruxolitinib is only about 25% [12]. Median survival is only 50 days in those who respond to additional agents, vs 21 days in those who do not [13].

Notably, there is no established third-line therapy for acute GvHD after the failure of ruxolitinib, and there is a lack of guideline-directed therapy in this setting. Limited data regarding surgical intervention in these patients who remain refractory exist, and data are largely confined to case studies [4,14].

Here, we present a case of glucocorticoid and ruxolitinib refractory lower GI GvHD following a hematopoietic stem cell transplant (HSCT) in a 41-year-old woman. Robotic total abdominal colectomy was offered as a salvage procedure after the failure of multiple immunomodulating agents and a hospital stay of nearly five months. After colectomy, her enteric symptoms resolved, and she was able to be discharged.

## Case presentation

A 41-year-old female underwent HSCT for stage IV angioimmunoblastic T-cell lymphoma with a myeloablative regimen consisting of cyclophosphamide and 8 Gy of total body irradiation from an 8/8 HLA-matched donor using post-transplant cyclophosphamide, tacrolimus, and mycophenolate mofetil for GvHD prophylaxis. She developed acute GvHD less than a month following engraftment. This presented with pseudomembranous conjunctivitis and diffuse erythematous maculopapular rash. Unfortunately, while on a steroid taper for resolving skin GvHD, she developed profuse watery diarrhea and was admitted to the transplant service for intravenous corticosteroids, initiation of ruxolitinib, and enteric rest with parenteral nutrition.

**Figure 1:**
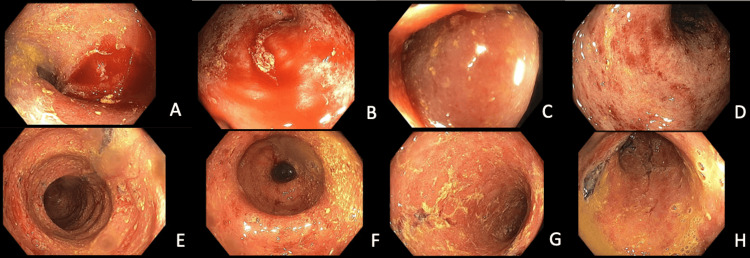
Colonoscopy shortly following admission Moderate to severe inflammation appreciated from the terminal ileum (A) and cecum (B, C), extending throughout the colon (D-G) and rectum (H)

A colonoscopy performed shortly following admission was consistent with GvHD, demonstrating pancolitis with severe inflammation as well as terminal ileal (TI) involvement (see Figure 1). After her condition continued to worsen despite ruxolitinib, she was started on multiple additional therapies for GvHD including extracorporeal photopheresis, sirolimus, rituximab, lithium, budesonide, belumosudil, and vedolizumab. Her clinical picture continued to worsen, and ultimately, her GvHD progressed to grade IV disease with abdominal pain and bloody diarrhea. In addition, she developed altered mentation, pseudomonal pneumonia, and enterococcal bacteremia, requiring ICU level of care.

**Figure 2:**
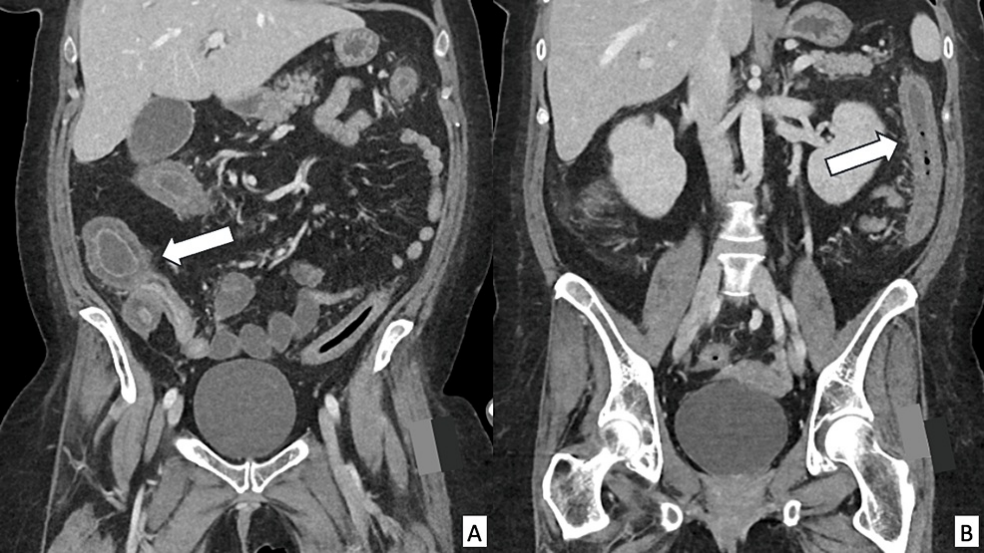
CT of the abdomen five months following admission Significant pan colonic mural thickening, including the cecum, terminal ileum (A), and descending colon (B)

**Figure 3:**
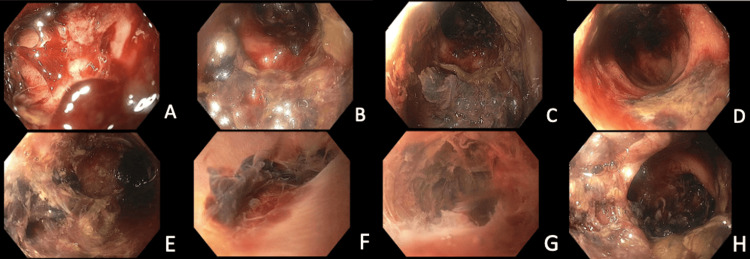
Flexible sigmoidoscopy 11 days prior to colectomy Severe inflammation and necrosis in the rectum (A-E) and sigmoid colon (F-H), procedure aborted due to observed tissue quality

Nearly five months following her admission, in the setting of ongoing severe bloody diarrhea and abdominal pain, CT imaging was obtained. This demonstrated TI and pan colonic wall thickening with submucosal edema and enhancement consistent with GvHD (see Figure 2). One week later, a flexible sigmoidoscope demonstrated severe inflammation and deep ulcerations. Endoscopy was aborted, and no biopsy was performed due to the poor quality of the tissue (see Figure 3).

Given her severe refractory disease to multiple agents (at the time, she was on two steroids, ruxolitinib, sirolimus, belumosudil, and photopheresis), colorectal surgery offered a salvage total abdominal colectomy. She was hemodynamically normal at this point, though with a persistent pancytopenia, including a hemoglobin of 8.8, white blood cell count of 1.4, and platelet count of 47. In light of these findings, a minimally invasive approach was pursued. On post-transplant day 172, this was completed robotically, with 20 cm of diseased ileum removed in continuity with her colon. The proximal resection margin was determined with a visual inspection of the exterior surface of the small bowel; this correlated well with the extent of disease appreciated on the aforementioned CT. Gross examination of the remaining small bowel did not reveal any further disease. At the rectosigmoid junction, the tissue appeared comparatively healthy and was thus divided, leaving most of the rectum in situ. An end ileostomy was created. Examination of the colonic specimen demonstrated an entirely effaced mucosal surface replaced by hemorrhagic granulation tissue.

Following surgery, the patient's diarrhea and abdominal pain resolved, and she was discharged following another episode of pseudomonal pneumonia. She was maintained on IVIG, sirolimus, and ruxolitinib.

## Discussion

Overall, the management of gut GvHD remains challenging, and surgery continues to be a last resort when other management strategies fail. Given the poor prognosis associated with lower GI GvHD, two retrospective studies have evaluated patients undergoing diverting loop ileostomy (DLI) for steroid refractory disease. In both studies, one with 13 [15] and one with 10 patients undergoing surgery [16], overall survival was improved in the DLI groups. Otherwise, literature regarding surgical intervention in GvHD is limited to case reports focusing on those who develop frank perforation or uncontrolled hemorrhage; as such, most documented cases are open procedures [17,18].

Surgery of any type poses clinical challenges in this patient population. Patients are frequently deconditioned and on immunomodulators that impair healing, putting them at greater surgical risk. While DLI may be appealing, as it is a less intensive procedure than a colectomy, if the disease remains unchecked in the colon, further complications may develop, including those that require additional surgery. Both the decision to operate and the choice of resection vs DLI mirror the surgical decision-making in Crohn's disease (CD), which, much like GvHD, affects a multitude of organ systems [19]. Fecal diversion is sometimes employed in recrudescent Crohn's colitis or perianal CD, with some cohort studies reporting symptom remission in over 50% of patients [20]. However, those with severe disease often require a colectomy; in these cases, operative intervention is often delayed, while some of the same immunomodulators used in GvHD are trialed [19]. In both CD and GvHD, surgery is often deferred, but may ultimately be required, in the sickest patients if they are to have a chance of recovery.

Here, surgery was only pursued as a salvage procedure as other management options had been completely exhausted. Given her deconditioning from prolonged hospitalization and high-dose steroid use, this procedure was exquisitely high risk, and the patient and her family were counseled to this effect. Given the poor tissue quality observed on colonoscopy, this approach was preferred to DLI. That the procedure was accomplished robotically was favorable in terms of her overall recovery and was feasible given her relative stability. At present, her intestinal symptoms have not returned, and evaluation for ostomy reversal may be considered in another six months if her clinical picture remains favorable.

## Conclusions

Gut GvHD is a potentially devastating complication of HSCT; while newer treatment options exist in the form of immunomodulating agents, patients who remain refractory may be considered for surgery if the disease extent is amenable to resection. While data are emerging on the role of DLI, resection for isolated colonic or TI involvement appears to be an appropriate alternative. Balancing the timing of surgery with the overall clinical picture is key, and experience gleaned from the management of CD can be of use here. 
